# Prescribing differences in family practice for diabetic patients in Germany according to statutory or private health insurance: the case of DPP-4-inhibitors and GLP-1-agonists

**DOI:** 10.1186/s12875-016-0543-7

**Published:** 2016-10-19

**Authors:** Gunter Laux, Sarah Berger, Joachim Szecsenyi, Petra Kaufmann-Kolle, Rüdiger Leutgeb

**Affiliations:** 1 Department of General Practice and Health Services Research, University Hospital Heidelberg, Voßstrasse 2, 69115 Heidelberg, Germany; 2 AQUA-Institute for Applied Quality Promotion and Research in Health Care, Göttingen, Germany

## Abstract

**Background:**

The objective of this study was to analyze prescription decisions for family practice (FP) patients with Diabetes mellitus type 2 (DM2) using the case of the incretin mimetics Dipeptidyl peptidase-4 (DDP-4) inhibitors and Glucagon-like peptide-1 (GLP-1) agonists dependent on patients’ health insurance status (statutory or private) in Germany. This study is important since the scientific debate is still open with regard to DPP-4-inhibitors and GLP-1-agonists, where some critics are raising questions on potential long-term risks for patients.

**Methods:**

Data for this analysis were sourced from the German health services research register CONTENT (CONTinuous morbidity registration Epidemiologic NeTwork), in which FP health services information, generated by family practitioners, is continuously collated, e.g. patients’ health insurance status, morbidity and pharmacotherapy. Patients with Diabetes mellitus type 1 (DM1) were excluded from the study.

**Results:**

From the family practices collaborating in the CONTENT research network, there were 7298 patients treated with pharmacotherapeutic agents for DM2 between 01.09.2009 and 31.08.2014. 586 (8.03 %) of these patients had private insurance. Prescriptions for the incretin mimetics were 40.6 % higher (9.7 vs. 6.9 %; *p* < 0.0001) for patients with private insurance compared to patients with statutory health insurance. This finding was confirmed with multivariable analyses.

**Conclusions:**

There was a statistically significant difference found in prescription patterns according to the patient’s health insurance status for the incretin mimetics in this sample population of German patients with DM2. Obviously, these differences result from the eligibility for reimbursement according to patients’ health insurance status. Whether incretin mimetics pose specific long term risks for particular patients is yet to be determined.

## Background

In the German health care system, there are two coexisting health insurance types (statutory or private) for individuals. These types differ in terms of contribution, access to health care services and reimbursement. The main differences are shown in Table 1.

**Table 1:** Characteristics of statutory and private health insurance in Germany

Characteristic	Statutory Health Insurance	Private Insurance
Principle of insurance	Principle of solidarity. Health care has to be economical and medically necessary. More than 90 % of German inhabitants have statutory health insurance.	Principle of equivalence. Policy of equivalence of service and reward. Less than 10 % of German inhabitants are privately insured. Self-employed persons and persons with an annual salary above 54,900€ have the option of private health insurance.
Insurance contribution	Health insurance contribution currently 14.6 % of the individual’s gross income. Additional contributions are possible.	Contribution dependent on age, sex, individual health risk and chosen services.
Access to health care system	Medical care of authorised physicians, authorized dentists and if possible inpatient treatment in the nearest hospital.	Free selection of physicians, dentists and hospitals.
Settlement of medical services	Billing of the medical services directly to the health insurance company.	Billing of the medical services directly to the patients, who apply afterwards for reimbursement from the private health insurance company.
Restrictions for pharmacotherapy and reimbursement (German Social Code, Book Five)	Limitation of budgets regarding patented medication. Prescription of generic drugs, if possible.	No restrictions.

The last row of Table 1 highlights that there are some restrictions for statutorily insured patients. In particular, differences in reimbursement for certain drugs can result in different prescribing decisions of family practitioners (FPs).

The objective of this study was to analyze this issue in a German family practice setting by examining FP prescription practices for patients with Diabetes mellitus type 2 (DM2) using the case of the incretin mimetics Dipeptidyl peptidase-4 (DDP-4) inhibitors and Glucagon-like peptide-1 (GLP-1) agonists.

The use of oral antihyperglycaemic agents for treating patients with DM2 is recommended when lifestyle interventions alone, including eating healthily, exercising regularly and losing weight, prove inadequate in maintaining blood glucose at target levels. In such cases, the oral biguanide, Metformin, is the first-line agent recommended in clinical guidelines [1]. Other agents should be considered whenever the use of Metformin is not possible or not sufficient.

These stepped recommendations are especially true for the “German National Guideline” for DM2. The current version of the guideline is very considerable and covers more than 250 pages [2]. The guideline is well structured and copes with the complexity associated with DM2. Both general recommendations and specialities important in DM2 disease management are considered adequately. Importantly, recommendations are given under consideration of existing national and international evidence. DPP-4 inhibitors and GLP-1 agonists are explicitly addressed in this guideline. Within the current version, both agents are classified as “antidiabetics without ascertained advantageous influence on clinical endpoints”. However, it is stated that there is no intrinsic risk of hypoglycaemia, neither for DPP-4 inhibitors nor for GLP-1 agonists. The DPP-4 inhibitor active agents “Sitagliptin” and “Vildagliptin” are permitted as monotherapy according to the current German guideline only if an intolerance to Metformin is observed. GLP-1 agonists should only be taken into account in combination with other oral antihyperglycaemic agents (preferably Metformin) for patients with severe weight problems, disposition to hypoglycaemia and/or cardiovascular problems, where Sulfonylurea are contraindicated.

Overall, there is currently a lack of consensus regarding the management of DM2 patients that fail to respond to initial monotherapy with metformin. New antihyperglycaemic agents such as DDP-4 inhibitors and GLP-1 agonists have entered the market and are being used either as a monotherapy or in addition to metformin [1]. As a result, when managing patients with DM2, the treating physician – commonly a FP – may now prescribe from an extensive range of pharmacotherapeutic agents. This includes the new classes of diabetic drugs such as DPP-4-inhibitors and GLP-1-agonists, which remain under patent protection and are therefore quite expensive. According to the German Diabetes Association (DDG), the primary advantage of DPP-4 inhibitors in comparison to the older sulfonylureas is the decreased risk of hypoglycaemia. An additional effect is a reduction in appetite and thus the reduced likelihood of weight gain [3]. These effects have been confirmed in various international studies on DPP-4-inhibitors and GLP-1-agonists [4]. Nevertheless, to date, there is little international evidence on the long-term risks for patients with these new classes of diabetic drugs.

Aside from taking into account therapeutic considerations and potential long-term risks, other factors such as eligibility for reimbursement and funding volumes steer German FPs when making prescribing decisions. When writing prescriptions, German FPs are expected to comply with requirements for cost-effectiveness (and in some cases prescription targets) set out in the guidelines on funding volumes from the statutory health insurance organisations [5]. However, cost-control restrictions on prescribing in this form do not exist for patients covered by private health insurance. Although there are legally required rebates on prescription drugs connected to patients with private health insurance, these have no specific financial implications for the prescribing FP.

Therefore, the object of this study was to examine FP prescribing patterns for the incretin mimetics DPP-4-inhibitors and GLP-1-agonists.

Of particular interest, while taking into account context-relevant covariates (age, sex, mortality, family practice), was the factor of patient health insurance status i.e. private or statutory and possible different prescription patterns of FPs according to the respective health insurance status.

## Methods

Data for this analysis were sourced from a German health services research Register, in which primary care health care information, generated by family practices, is continuously collated e.g. patients’ health insurance status (statutory or private), morbidity, disease progression and health care outcomes. This Register is overseen by the Research Network CONTENT (CONTinuous morbidity registration Epidemiologic NeTwork), which was established through a grant from the German Federal Ministry of Education and Research (BMBF) [6, 7]. Participation is voluntary for FPs. There are two software companies (namely “Quincy” and “S3”) supporting the CONTENT documentation that is based on both the ICPC (International Classification for Primary Care) and the ICD-10. Moreover, the software allows an automatized and secure data export to the central CONTENT database. For each patient, there is a unique pseudonym. Due to data protection regulations, the transmitted data do not contain patient identifying elements (e. g. patient names). For this study, patient data from 35 FPs were available, where contributions to the database for the whole observation period occured.

Uniquely for Germany, is that not only the records on prescriptions for statutorily health insured patients are available in the CONTENT register, but also those for privately insured patients. Furthermore, it is possible to differentiate between privately insured and statutory insured patients based on individual patient identifier codes. Prescription data is automatically collected in electronic patient records when the prescription is printed. Moreover, due to the central pharmaceutical numbers (“Pharmazentralnummer”, PZN), it is absolutely clear which medications and active pharmaceutical ingredients have been prescribed.

Data from 7298 patients from a 5-year cohort (01.04.2009 to 31.08.2014) that was generated by family practices collaborating in the CONTENT research network served as the basis for this analysis. These patients were treated with pharmacotherapeutic agents for DM2. Patients with Diabetes mellitus type 1 (DM1) were excluded from this analysis. Total morbidity was calculated using the Charlson Index [8], in which clinical conditions based on ICD-10 diagnoses were scored and scores were summed to provide a total morbidity score.

A written informed consent from patients was not obtained. In Germany, anonymized patient data can be used without written informed consent, if the data are used for strictly scientific purposes.

In order to determine whether patient health insurance status i.e. private or statutory was a statistically significant influencing factor, univariate analyses of differences between privately insured and statutory insured patients were made using Fisher’s exact test [9]. Multivariable analyses [10] were performed using binary logistical regression under adjustment for age, sex and total morbidity of patients. In addition, a two-level (doctor, patient) analysis was conducted. Statistical software used for these analyses were R (Version 3.1, 64 bit) [11] und SAS (Version 9.4, 64 bit) [12]. Ethical approval for the CONTENT project was given by the University Hospital Heidelberg Ethics Committee (No. 442-2005).

## Results

From the family practices collaborating in the CONTENT research network, there were 7298 patients treated with pharmacotherapeutic agents for DM2 between 01.09.2009 and 31.08.2014. 594 (8.02 %) of these patients had private insurance. Although average age was very similar across both groups, the percentage of females in the group with statutory health insurance was much higher. In addition, the total morbidity score, according to the Charlson Index, was higher in the statutory health insurance group reflecting a higher health care burden (Table 1).

The average number of antidiabetic prescriptions per patient was 12.1 ± 15.7 for statutorily and 11.9 ± 14.6 for privately insured patients (p: n.s.) within the observation period. Prescriptions for the incretin mimetics were 40.6 % higher (9.7 vs. 6.9 %; *p* < 0.0001) for patients with private insurance compared to patients with statutory health insurance. Equally, when DPP-4-inhibitors and GLP-1-agonists were analyzed separately, there remained a statistically significant difference. A particular non incretin mimetic antidiabetic therapy was totally replaced by an incretin mimetic therapy for 34.2 % of statutorily insured patients and for 54.7 % of privately insured patients. For 65.8 and 45.3 % of patients, respectively, an incretin mimetic therapy was added. The observed difference was statistically significant (*p* < 0.0001).

The univariate correlation between type of health insurance and prescription for incretin mimetics (OR = 1.44; 95 %-CI:[1.32, 1.57]) was confirmed with a 2-level (patient, practice) multivariable analysis (Table 2) using context relevant covariates (age, sex, total morbidity) (OR = 1.39; 95 %-CI:[1.24, 1.55]; *p* < 0.0001).

**Table 2:** Prescription patterns for incretin-mimetics with analyzed covariables

	Patients with statutory health insurance *n* = 6712 (92.0 %)	Patients with private health insurance *n* = 586 (8.0 %)	Statistical Significance (p-Wert)
Average age in years (± SD)	69.9 ± 12.1	69.2 ± 12.1	n. s.
Sex (% female)	53.8	31.1	*p* < 0.0001
Morbidity (± SD)	1.92 ± 1.78	1.44 ± 1.67	*p* = 0.0008
Percentage of prescriptions for DPP-4 inhibitors (%)	6.3	8.4	*p* < 0.0001
Percentage of prescriptions for GLP-1 agonists (%)	0.6	1.3	*p* < 0.0001
Percentage of prescriptions for incretin-mimetics (%) (DPP-4-inhibitors or GLP-1-agonists)	6.9	9.7	*p* < 0.0001
Percentage of patients with at least one incretin-mimetic prescription in the observation period (%)	15.3	21.0	*p* < 0.0001
For these patients, in the observation period, there were also prescriptions for			
• Insulin, incl. premixed and analogues (%)	24.8	15.4	*p* = 0.0245
• Metformin as monotherapy (%)	69.9	61.0	*p* = 0.0497
• Sulfonylurea as monotherapy (%)	30.4	14.6	*p* = 0.0002

Under consideration of the mentioned covariables, the “chance” for privately insured patients with DM2 to be prescribed incretin mimetics was significantly and relevantly higher in comparison to patients with statutory health insurance in this sample (Tables 2 and 3).

**Table 3:** Result of multivariable analysis

Covariable	Odds Ratio [95 %-CI]	Statistical Significance (*p*-Value)
Age in Years	0.983 [0.981, 0.985]	*p* < 0.0001
Sex (0: male, 1: female)	0.870 [0.826, 0.917]	*p* < 0.0001
Morbidity (Charlson-Index)	0.998 [0.920, 1.079]	n. s.
Health insurance (0: statutory, 1: private)	1.385 [1.240, 1.546]	*p* < 0.0001

## Discussion

Different health insurance types in Germany are associated with particularly different health care service restrictions for the patients. This is especially true in terms of pharmacotherapy. In this study, we observed statistically significant differences in prescribing of the incretin mimetics DPP-4-inhibitors and GLP-1-agonists in the sample population for patients with DM2 in family practice. This analysis cannot prove a causal relationship between differences in eligibility for reimbursement according to private or statutory health insurance and prescription patterns for these new classes of diabetic drugs. However, it is certainly strongly suggestive that this is the case.

Other data sources show that since these new classes of diabetic drugs have entered the German market there is a noteworthy associated flow of finances, at least in the private insurance sector. According to results published in the German major 2012 report on pharmacotherapeutic agents for privately insured patients, a steady increase in prescriptions for incretin mimetics to the sum of 45.6 M € (+108.7 %) was reported for the previous 5-year timeframe. This included DPP-4-inhibitors in combination with metformin, GLP-1-agonists and also insulin analogues, which reflects as well the aging population [13].

According to the German federal health insurance law, FPs are required to prescribe statutory insured patients mimetics when possible and to prescribe new agents in a reserved manner [14]. Additionally, pharmacies are committed to dispensing drugs from discount contract drug suppliers from the patient’s health insurance company [15]. FPs overriding their prescribing funding volume are threatened by a reclaim of drug costs from the insurance companies.

In the context of special health insurance company contracts, FPs have to attend quality circles offered independently of pharmaceutical company sponsoring. During these quality circles, current studies and guidelines are presented and cost-saving potentials are considered. Feedback reports of the prescriptions of individual FPs in comparison to all the FPs of one region are generated.

Currently, Germany is facing a tremendous increase in the prescriptions of incretin based antidiabetics. This is due to rigorous marketing of the manufacturers addressing primarily FPs, internists and diabetologists. Incretin based drugs are now contributing a major share of total costs from oral antidiabetic drugs [16]. In our sample, we could observe a rise for the prescription rate for both statutorily and privately insured patients over time. However, the prescription rate for incretin mimetic was about 40 % higher for privately insured patients in every observation year.

In the international literature, the debate is still open with regard to DPP-4-inhibitors and GLP-1-agonists, where some critics are raising questions on potential long-term risks for patients. The 2013 critical analysis by Butler et al. [4] from the University of California made a summary of recent studies, which recognised the benefit of the decreased risk of hypoglycaemia, but also highlighted potentially disadvantageous long-term effects. Of particular concern were cases of acute and chronic pancreatitis as well as malignancies in the pancreas and thyroid. Other literature comes to similar conclusions, but these were results of rodent studies [17, 18]. Nevertheless, in the recently published 2014 meta-analysis from Li et al. [19] and an assessment co-published by the Food and Drug Administration und the European Medicine Agency no causal relationships could be proven between pancreatitis and incretin mimetics [20]. A similar conclusion has been drawn by Nauck et al. [21], who correctly point out that owing to the modest amount of data available, it is not possible to completely exclude possible risk.

The European Medicines Agency (EMA) is currently examining new data on the DPP-4-inhibitor “Saxagliptin” in terms of mortality since the US-American FDA (Food and Drug Administration) observed an association between the intake of Saxagliptin and heightened mortality in the context of the “SAVOR” study [22].

Recently the FDA warned that DPP-4 inhibitors for type 2 diabetes may cause severe joint pain [23]. On the other hand, some studies indicate that cardiovascular side effects with the DPP-4 inhibitors are less frequent in comparison to other antihyperglycaemic agents, although it cannot be taken as far as to claim they provide a protective cardiovascular effect [24–26]. And finally, new results of the TECOS study demonstrate that the DDP-4 inhibitor agent “Sitagliptin” reduced the risk of initiating an additional antihyperglycemic agent during the study by 28 %. “Sitagliptin” was comparable to other antihyperglycemic agents regarding the incidence of severe hypoglycemia and the use of this agent had a neutral effect on all cardiovascular outcomes [27, 28].

When FPs are making prescribing decisions for patients with DM2, aside from taking into account therapeutic considerations and advantages of the incretin mimetics, it needs to be also taken into account that potential long-term risks are as yet unclear. As a classic lesson, there is the *“Glitazones”* class of diabetic medications that in some cases have been withdrawn completely from the market and in other cases are no longer recommended due to concerns of increased incidence of coronary heart disease and myocardial infarction or possible links to bladder cancer associated with their use [29, 30]. Currently there is still disagreement between different expert associations regarding the potential therapeutical advantage of the GLP-1 and DDP-4 agents and the potential risks and side effects of such a therapy [31, 32].

Critical reflection and reference to clinical guidelines and current literature belongs to good medical practice when making prescribing decisions and this is equally relevant for prescription of DPP-4-inhibitors and GLP-1-agonists, the case under discussion in this paper. It certainly has to be recognised that with more or less free prescribing in Germany for privately insured patients of new classes of diabetic drugs such as the incretin mimetics, these patients have a potential therapeutic advantage over patients with statutory health insurance due to easier access. However, it should be emphasized that in all cases, good medical practice for prescription decisions related to DPP-4-inhibitors and GLP-1-agonists should be based on potential therapeutic advantages and potential disadvantages/risks of the pharmacotherapeutic agents and not eligibility for reimbursement according to private or statutory health insurance.

The strength of this study include the ability to compare data from patients with either private or statutory health insurance receiving primary health care services from the same FP, due to information being continuously collated in a health services research Register from the family practices collaborating in the CONTENT research network. In contrast to other known German registers such as DiaRegis [33] or SIRTA [34], our Register was not explicitly established to investigate research questions related to DM2. Data from this Register provides a comprehensive overview of multiple health issues and their treatments. Currently, the Register has collected morbidity and health services data from a total of 3M Doctor-Patient contacts. The Research Network CONTENT has much future potential in terms of synergistic effects, in cooperation with other existing registers, to address research needs and produce evidence with a focus on primary care health services by FPs for patients with DM2.

Limitations related to this study include the use of routine data collected from family practices collaborating in the CONTENT research network. Data on prescriptions made by specialists (particularly Internal Medicine) were not available. In addition, other factors taken into account in therapeutic decision-making beside the socio-demographic data (e.g. occupation, leisure activities, driving) were not available in the register, and could be relevant. Moreover, is has to be taken into account that the data was derived from voluntarily participating FPs within a regional German cluster (mainly Baden-Württemberg and Hesse, 2 of 16 federal states of Germany). These factors need to be taken into consideration in terms of the representativeness of the results.

## Conclusions

In this sample population of German patients with DM2, we observed statistically significant differences in prescription patterns according to the patient’s health insurance status for the incretin mimetics. This is clearly due to differences in the eligibility for reimbursement according to patients’ health insurance status. Of concern, is the fact that whether incretin mimetics pose specific long term risks for particular patients is yet to be determined. In conclusion, whether a patient has private or statutory health insurance should not determine pharmacotherapeutic advantages or risks for patient groups with a particular health problem. This needs to be taken into account by key stakeholders and decision-makers in the development of new strategies and measures in health care service provision.

## Abbreviations

BMBF: “Bundesministerium fuer Bildung und Forschung ” (Federal Ministry of Education and Research)
CI: Confidence Interval
CONTENT: CONTinuous morbidity registration Epidemiologic NeTwork
DDP-4: Dipeptidyl peptidase-4
DM1: Diabetes mellitus type 1
DM2: Diabetes mellitus type 2
EMA: European Medicines Agency
FDA: Food and Drug Administration
FP: Family Practitioner
GLP-1: Glucagon-like peptide-1
n. s.: not significant
OR: Odds Ratio
